# Repurposing Plant Virus Nanoparticles

**DOI:** 10.3390/vaccines6010011

**Published:** 2018-02-14

**Authors:** Kathleen L. Hefferon

**Affiliations:** Department of Food Sciences, Cornell University, Ithaca, NY 14886, USA; klh22@cornell.edu; Tel.: +1-607-387-6304

**Keywords:** plant virus, nanoparticle, epitope, vaccine, cancer, imaging

## Abstract

Plants have been explored for many years as inexpensive and versatile platforms for the generation of vaccines and other biopharmaceuticals. Plant viruses have also been engineered to either express subunit vaccines or act as epitope presentation systems. Both icosahedral and helical, filamentous-shaped plant viruses have been used for these purposes. More recently, plant viruses have been utilized as nanoparticles to transport drugs and active molecules into cancer cells. The following review describes the use of both icosahedral and helical plant viruses in a variety of new functions against cancer. The review illustrates the breadth of variation among different plant virus nanoparticles and how this impacts the immune response.

## 1. Introduction

Plants as bioreactors for modern pharmaceutical products have been under investigation for many years, and these are only now beginning to hit the marketplace. From microbicides for HIV treatment, vaccines for pandemic influenza, to a therapeutic agent for Gaucher’s disease, plant-made pharmaceuticals are being readily employed for a variety of purposes in medicine today [1,2,3]. Plant-made vaccines lack the safety considerations that are associated with conventional vaccines and cannot be subject to contamination by human pathogens, are easy to upscale, cheap, and lack refrigeration requirements as well as a sophisticated medical infrastructure [4,5]. These attributes have provided plant-made pharmaceuticals with an opportunity to assist those in resource poor settings to receive medicines that were previously inaccessible [6].

Plant viruses have also undergone research and development for their potential use as expression vectors for pharmaceutical production [4,5]. Plant viruses can produce large amounts of heterologous proteins within a very short time frame, and lack the same controversial public perception associated with the production of transgenic plants. Plant viruses can both produce full-length proteins for pharmaceutical uses, such as the monoclonal antibody for Zika virus [7]. Alternatively, short epitopes can be expressed on the outer surface of the capsid protein in such a way that assembled virus particles act as vehicles for epitope display [8]. While full-length virus expression vectors were originally employed for vaccine production, recently, the use of deconstructed virus vectors, such as the magnICON system, have been utilized for ease of use, to achieve maximum expression levels, and to maintain high levels of biocontainment (deconstructed vectors lack the genes needed for virus transmission or cell-to-cell movement, and thus avoid concerns of escape into the environment) [9,10]. 

Over the past few years, plant viruses have found a novel role as nanoparticles, to not only deliver medical materials such as drugs, but also sources of material for electronics and optics, as examples [11,12,13]. It is striking to note that several plant viruses have been shown to accumulate at solid tumors and elicit a highly localized immune response within the surrounding microenvironment [14]. As plant virus nanoparticle systems improve in sophistication, they are being examined for their ability to activate the T cell response, and thus represent alternative immunotherapy strategies for cancer and other chronic diseases [14]. They are safe, stable in vivo, biodegradable, and easy to produce. While nanoparticles based on plant viruses do not cause adverse reactions, and yet can be highly immunogenic, they have so far only been tested on preclinical models. These characteristics make plant virus nanoparticles prospective vehicles for use in developing countries where patients have low access to modern medical treatment and thus poor prognosis for survival. 

Plant viruses are frequently characterized by their icosahedral or helical morphologies; these differences offer a range of attributes which make them suitable for a variety of purposes in medicine as well as in engineering [11,12,13]. Both morphologies can carry drugs or imaging reagents that are conjugated to the outer surface of the capsid protein, thus producing a highly regular repeating pattern on the surface of the virus particle [12]. The helical shape of some plant viruses appears to assist them in their ability to home in on solid tumors [11]. Icosahedral viruses, on the other hand, can carry reactive molecules such as drugs within their internal cavity and release them under the appropriate physiological conditions [15,16,17]. The following review describes the incorporation of icosahedral and helical plant viruses as immunotherapy agents to assist in the battle against chronic diseases, including cancer. 

## 2. Icosahedral Virus Nanoparticles 

**Figure 1:**
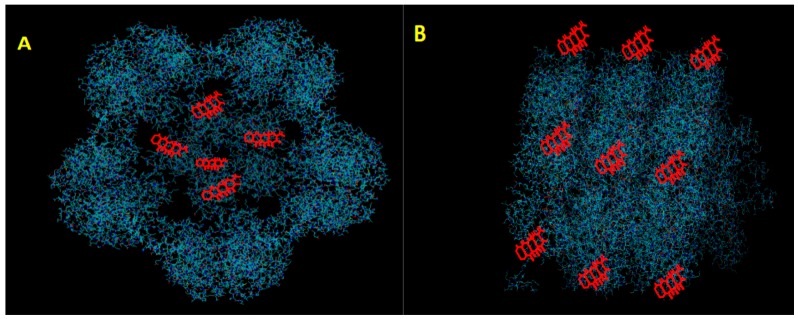
Schematic diagram of plant virus nanoparticles associated with Doxorubicine (DOX). In this diagram, DOX is oversized for visualization. (**A**) DOX residing within internal cavity of an icosahedral virus (**B**) DOX conjugated to the outer surface of a helical virus.

Plant viruses which possess an icosahedral morphology and have been engineered as nanoparticles include Cowpea mosaic virus (CPMV), Brome Mosaic virus (BMV), Red clover necrotic mosaic virus (RCMNV), Hibiscus chlorotic ringspot virus (HCRSV), Johnson grass chlorotic stripe mosaic virus (JgCSMV), and Physalis Mottle Virus (PhMV) (Table 1). The highly ordered symmetry of icosahedral viruses provides a multivalent scaffold for eptiope display [18]. Conversely, empty virus-like particles based on icosahedral plant viruses offer a molecular entrapment platform for carrying drugs, imaging agents and other materials [19]. Drugs can be loaded into icosahedral plant viruses through covalent attachment to certain reactive moieties on the capsid protein. Loading the interior cavity of a VLP can take place through a gated mechanism that is sensitive to pH and metal ion concentration (Figure 1). For example, at high pH, some VLPs are induced to swell and open pores so that a drug cargo can enter. When the pH is lowered again, the swelling is reversed and the drug is trapped inside. These icosahedral nanaparticles can be targeted through the conjugation of a variety of tissue-specific ligands to the exterior of the virus particle [17]. Examples of the current use of icosahedral plant virus nanoparticles are described below. 

Of all the icosahedral plant viruses that have been developed as nanoparticles, Cowpea mosaic virus (CPMV) has been studied the most extensively. CPMV has been found to bind specifically to vimentin, which is expressed on the surface of many types of cells, including HeLa cells. Upon binding, CPMV can be internalized by endocytosis [38,39]. Uptake of CPMV by antigen-presenting cells enables it to block a number of cancers by direct stimulation of the immune system [20]. More recently, empty CPMV virus-like particles (eVLPs) have been able to self-assemble in the absence of genomic RNA and, when applied to a tumor, were able to alter the surrounding microenvironment to potentiate tumor immunity [20]. Since the immune response is highly localized, the chance of patients encountering an adverse reaction to this type of treatment is low.

A mouse model has been used to demonstrate that CPMV nanoparticles can act as effective immunotherapies for lung metastatic melanoma models and other cancers by inducing an innate immune cell-mediated anti-tumor response [22]. CPMV nanoparticles that were added to bone marrow-derived dendritic cells (DC) were able to increase proinflammatory cytokine level expressed by DCs. CPMV nanoparticles that were administered via inhalation dramatically increased neutrophil populations within twenty-four hours, and tumors could be reduced through weekly injection with the nanoparticles, suggesting that the activation of neutrophils invokes the CPMV-directed anti-tumor effect in colon, breast and ovarian cancer model systems.

Madden et al. (2017) examined the doxorubicin containing Red clover necrotic mosaic virus (RCNMV) as a drug delivery vehicle. The effectiveness RCNMV nanoparticles (PVNs) were compared with PEGylated liposomal doxorubicin (PLD) and small molecule doxorubicin using mouse ovarian cancer and melanoma models [23]. The authors found that the plant virus nanoparticles worked more efficaciously than the PEGylated liposomal doxorubicin in the ovarian cancer model, but not in the melanoma cancer model. The plant virus nanoparticles were cleared from the plasma more rapidly, however, they were delivered more efficiently to tumors than PLDs, demonstrating that they can be superior delivery vehicles for certain cancer types. 

Another example is Hibiscus chlorotic ringspot virus (HCRSV), a positive-sense, RNA icosahedral virus 30 nm in diameter and of the family Tombusviridae. The coat protein of HCRSV is 38 kDa and can self-assemble into empty virus-like particles. The authors Ren et al. (2007) first demonstrated the ability of HCRSV to transport drug molecules polystyrenesulfonic acid (PSA) and polyacrylic acid (PAA) [24]. The authors also conjugated VLPs with folic acid (a targeting molecule) and encapsulated the anticancer drug doxorubicin, to demonstrate that this delivery system could elicit cytotoxicity in human ovarian cancer cells [18]. 

Alemzadeh et al. (2017) used Johnson grass chlorotic stripe mosaic virus (JgCSMV), a member of the Tombusvirus family, to produce empty virus-like particles [25]. JgCSMV is a 30 nm icosahedral shaped virus with a capsid protein of 41 kDa. Both infectious particles containing genomic RNA and empty shells are found in infected plants. The virus-like particles that are generated are extremely stable. These VLPs were used as nanocarriers for the anti-cancer drug doxorubicin (DOX). The authors expressed empty CP in a tobacco hairy root expression system and demonstrated that the anti-cancer drug doxorubicin DOX can be loaded into the virus particles and produced at high quantities. 

Physalis mottle virus is a 30 nm icosahedral member of the tymovirus family, with a positive sense RNA genome. The 21 kDal CP can self-assemble in *E. coli* to make *p* = 3 structured virus-like particles that resemble the wild type virus. Masarapu et al. (2017) found that the N-terminus of the CP can be modified without deleterious effect to virus assembly [26]. VLPs derived from PhMV are stable and robust, and can be grown to very high concentrations in plants. PhMV nanoparticles can be functionalized to carry cancer drugs doxorubicin (DOX) and mitozantrone (MTX). For example, the authors used PhMV conjugated to the fluorophore Cyt-5 to demonstrate that virus nanoparticles were efficiently internalized into breast, ovarian and prostate cancer cells. The authors were able to determine whether these virion encapsidated drugs were able to retain their cytotoxic activity by comparing their action with cells treated with free, unencapsidated drugs. They found no significant difference in cytotoxicity between cells treated with free and encapsidated drugs. 

## 3. Helical Virus Nanoparticles

Helical, or filamentous virus nanoparticles are characterized by having a high aspect ratio, which enables them to be utilized for a whole variety of purposes and can include the tombusviruses such as Tobacco mosaic virus (TMV), the potexviruses such as Potato virus X (PVX) and Papaya mosaic virus (PapMV), and potyviruses such as Zuchinni yellow mosaic virus (ZYMV) [12,13] (Table 1). Their rod shape has enabled helical plant viruses to function as biotemplates for novel nanostructured material production, and knowledge of the interior and exterior cavities has assisted in the accurate incorporation of different moieties [12]. The differences in rigidity of TMV and flexibility of PVX rod-shaped particles, for example, has provided multiple options for material production at the nanoscale level, ranging from nanowires to biocatalysts [13]. The following section provides a few of these innovations as they relate to the field of medicine.

One powerful use for plant virus nanoparticles has been found in the field of personalized medicine. Non-Hodgkin’s Lymphoma (NHL) is a common hematologic malignancy with approximately one quarter of all cases presenting as follicular B-cell lymphoma. Conventional treatment for NHL include chemotherapy, radiation, passive antibody therapy and more recently, active immunotherapy [40]. Malignant B cells express a surface idiotype that is unique for that individual patient. If screened early enough, patients can be vaccinated based on their own idiotype; however, this procedure is expensive and time sensitive. Plant-made personalized vaccines against NHL are low in cost, rapid and relatively easy to implement. Plant-made NHL vaccines have been generated in Tobacco mosaic virus-based expression systems, and have ranged from a scFv subunit to a full-length immunoglobulin [41,42]. These are currently undergoing human clinical trials, and so far, the plant-made vaccines closely resemble vaccines made using other production platforms [27,28]. 

TMV has also been engineered as a drug carrying nanoparticle to combat non-Hodgkin’s lymphoma. Kernan et al. (2017) conjugated the antimitotic drug valine-citrulline monomethyl auristatin E (vcMMAE) targeting non-Hodgkin’s lymphoma, to TMV nanoparticles [29]. The authors demonstrated that each of the coat proteins of TMV carried vcMMAE. The nanoparticles were taken up by Karpas 299 cells, trafficked to the endolysosomal compartment, likely allowing for the release of the active monomethyl auristatin E component through the protease-mediated cleavage of the valine-citrulline linker. 

Jobsri et al. (2015) used Potato virus X nanoparticles containing on their surface idiotypic tumour antigens to elicit an immune response against murine B cell lymphoma [33]. The authors found that the viral ssRNA genome behaves as an adjuvant via Toll-like receptor 7 (TLR7) engagement to stimulate α-IFN secretion. 

Plant-derived vaccines have also been explored as a potential immunotherapy against solid tumors. For example, the TMV MagnICON system has been used to elicit a strong response against breast cancer, by expressing a fragment of Her2 [30]. PVX nanoparticles have been used to induce anti-Her2 antibodies with Herceptin-like properties (Trastuzumab), and induce apoptosis in breast cancer cell lines. Lee et al., 2017, examined the ability of PVX and DOX to delay tumor progression. The authors found that co-administered PVX and DOX worked better than PVX nanoparticles that were loaded with DOX, suggesting that it may not be necessary to coordinate chemotherapy and stimulation of the immune system using a single virus nanoparticle. The authors thought that since PVX slowed tumor growth, than PVX and DOX combined would act synergistically, but this did not appear to be the case [43]. 

The externally exposed side chains of the capsid protein of Potato virus X includes a lysine residue which can be functionalized to conjugate drugs or imaging reagents [44]. These virus nanoparticle conjugates could be further stabilized by PEGylation, and penetrate deeply within solid tumors to accumulate in the center core [15,45]. The potexvirus Papaya mosaic virus (PapMV) has also been examined for its immunostimulatory properties. Even without alteration, PapMV elicits an α-IFN-dependent response and can slow down melanoma progression in animal models [46,47]. Mice treated systemically with PapMV followed by B16-OVA cells 6 h later exhibited a reduced number of tumor nodules compared to untreated mice used as controls [34,35,36]. 

Carignan et al. (2017), demonstrated that Papaya mosaic virus (PapMV) nanoparticles containing single-stranded RNA could function as a TLR7 agonist and could be employed as an immune modulator in cancer immunotherapy [37]. The authors also demonstrated that stimulation of human peripheral blood mononuclear cells (PBMC) with PapMV induced the secretion of α-IFN and other pro-inflammatory cytokines and chemokines, including IL-6. Plasmacytoid dendritic cells (pDCs) became activated and promoted the NK cytolytic activity against cancer cells when co-cultured with NK cells. The authors conclude that PapMV displays multiple characteristics of being a viable a cancer immunotherapy agent. Plant potyviruses have also been used to reduce tumor progression. [48]. 

TMV can be further modified to be tumor-specific by functionalizing a tumor-homing peptide, such as cRGD, to its surface; this enables the virus to become internalized into tumor cells [49]. TMV nanoparticles have been engineered to treat ovarian cancer. Ovarian cancer is one of the leading causes of cancer-related death in women, mainly due to its late-stage diagnosis. Plantinum-based chemotherapy is used as a treatment; however, this can often lead to recurrence of the cancer due to platinum resistance, and death within a few years afterword. Cisplatin is the drug most commonly used for ovarian cancer. Franke et al. (2017) loaded the interior channel of TMV with cisplatin, a drug frequently used to treat ovarian cancer, to create TMV-cisPt [31]. By creating a simple ‘click’ reaction through incubation in an aqueous buffer, positively charged cisplatin was able to interact with negatively charged glutamine found on the TMV inner channel. The authors found that TMV was readily taken up into epithelial ovarian cancer cells. TMV-cisPt is both easy to produce and a low environmental hazard, making it an attractive new potential treatment for platinum resistant ovarian cancer.

TMV has also been used as a hollow nanotube to encapsulate with photosensitizers based on porphyrin dynamic sensors. The nanotubes were engineered to target melanoma cells and deliver their cargo. Photodynamic therapy (PDT) based on reactive oxygen species created during exposure to activation by specific light wavelengths, was used to efficiently kill the melanoma cells [32]. 

## 4. Other Applications for Plant Virus Nanoparticles

Besides vaccine and epitope carrying and drug delivery systems, other uses for plant virus nanoparticles have included an assortment of biosensors, optoelectronic devices, catalysts and imaging systems [11,12]. 

Plant virus nanoparticles are broad in application. For example, Pitek et al. (2017) demonstrated that Tobacco mosaic virus can home in to thrombi and deliver streptokinase via PEG linkers to mouse models of thrombotic cardiovascular disease [50]. The authors found that thrombolysis was enhanced, and speculated that this is due to the shape-mediated flow properties of TMV. Plant virus nanoparticles thus offer promise as a thrombolytic therapy [51]. Similarly, Dickmeis et al. (2017) took advantage of the filamentous properties of Potato virus X to present mineralization-inducing (MIP) and integrin binding motif (RGD) peptides on its surface. Virus particles isolated from plants were used to support hydroxyapatite crystal nucleation, indicating that plant virus helical nanoparticles show potential for bone tissue substitutes [52].

The flexuous rod-shaped virions of potyvirus Turnip mosaic virus (TuMV) has also been examined for peptide display. Recently, González-Gamboa et al. (2017) found that a peptide derived from the human thrombin receptor (TR) inhibited infectivity of recombinant TuMV. The authors produced virus-like particles using both wild type as well as recombinant capsid protein [48]. These VLPs were able to increase the amount of human thrombin receptor recognized by antibodies, demonstrating that the use of assembly of VLPs in plants can circumvent the problem of peptide insertion which reduces virus expression vector infectivity. 

More engineering strategies are being made to further enhance the utilization of TMV as a nanoparticle in medicine. Fischer et al. (2017) expressed the monomeric non-oxygen-dependent fluorescent protein iLOV to the C-terminus of the coat protein of TMV using an N-terminal Foot-and-mouth disease virus (FMDV) 2A ribosome skipping sequence [53]. Fluorochromes have been conjugated to plant virus nanoparticles so that they can be used as imaging vehicles. For example, virus nanoparticles based on PVX and CPMV have been engineered as highly specific cancer imaging reagents through conjugation with polyethylene glycol (PEG) polymers and a number of different targeting ligands [54,55]. 

The cysteine side chains of Potato virus X (Cys 121) have recently been identified as reactive groups for modification in addition to lysine [56]. Lysines were conjugated with Gd-DOTA for magnetic resonance imaging (MRI) and cysteines were conjugated with fluorescent dyes, in such a way that the nanoparticle could function in dual-modal optical-MRI imaging applications. 

## 5. Conclusions 

Over the past two decades, plant viruses are well on their way to becoming established as expression systems for vaccines and other pharmaceutical proteins. More recently, plant viruses have found a place in the fight against cancer, by acting as ‘smart’ nanoparticles that can home in on tumor cells and deliver drugs as cargo, as well as in many cases elicits a highly localized and powerful immune response [44]. Plant virus nanoparticles are inexpensive and easy to upscale, safe to use, have low toxicity and are biodegradable. Importantly, these properties give plant virus nanoparticles a potential role for treating chronic diseases in resource poor settings of the developing world.

Their inherent ability to penetrate solid tumors provides plant viruses with an added edge in our current arsenal of nanocarriers for cancer therapy [14,33]. Drugs can be functionalized to the surface of plant viruses, enabling them to provide many more tasks than mere epitope presentation. To date, plant virus nanoparticles have demonstrated clear efficacy on different pre-clinical cancer models ranging from lymphomas to breast cancer [57,58]. A variety of both plant viruses fully equipped with their genomes intact, as well as empty virus-like particles that lack their genetic material, have been shown to function in different settings [59,60]. As this newly emerging field continues to blossom, it is interesting to note that different plant viruses elicit different types of immune responses, signifying that the development of new virus nanoparticle systems ought to continue [61]. The outcome of this approach will be that many more features of plant viruses as elicitors of the immune response and as pharmaceutical carriers will become fully realized. Plant viruses have already found many uses in electronics, catalysis, imaging and even as pesticides. The uses of nanoparticles based on plant viruses will continue to expand for many years to come.

## Figures and Tables

**Table 1:** Select examples of applications for plant virus nanoparticles.

A. Icosahedral Viruses		
Plant Virus Nanoparticle	Application Tested	Reference
Cowpea mosaic virus (CPMV)	Solid tumors	[20,21,22]
Red clover necrotic mosaic virus (RCNMV) encapsulating DOX	Ovarian cancer, melanoma	[23]
Hibiscus chlorotic ringspot virus (HCRSV) encapsulating DOX, PSA and PAA	Ovarian cancer	[24]
Johnson grass chlorotic stripe mosaic virus (JgCSMV) encapsulating DOX		
Physalis mottle virus (PhMV) encasiulating DOX and MTX	Untested	[25]
**B. Helical Viruses**		
Tobacco mosaic virus (TMV) immunoglobulins, also nanoparticle carrying vcMMAE, Her2, cisplatin, porphyrin dynamic sensors	Breast, ovarian, prostate cancers	[26]
Potato virus X (PVX)		
Papaya mosaic vírus (PapMV)	Non Hodgkins Lymphoma, Ovarian cancer cells, melanoma cells	[27,28,29,30,31,32]
	Murine B cell lymphoma, Breast cancer cells	[30,33]
	Ovarian cancer cells	[34,35,36,37]
